# Polo-like kinase 1 (PLK1) O-GlcNAcylation is essential for dividing mammalian cells and inhibits uterine carcinoma

**DOI:** 10.1016/j.jbc.2023.102887

**Published:** 2023-01-07

**Authors:** Sheng Yan, Bin Peng, Shifeng Kan, Guangcan Shao, Zhikai Xiahou, Xiangyan Tang, Yong-Xiang Chen, Meng-Qiu Dong, Xiao Liu, Xingzhi Xu, Jing Li

**Affiliations:** 1 Beijing Key Laboratory of DNA Damage Response and College of Life Sciences, Capital Normal University, Beijing, China; 2 Guangdong Key Laboratory for Genome Stability & Disease Prevention and Carson International Cancer Center, Marshall Laboratory of Biomedical Engineering, Shenzhen University School of Medicine, Shenzhen, Guangdong, China; 3 Zaozhuang Municipal Hospital, Shandong, China; 4 National Institute of Biological Sciences, Beijing, China; 5 Key Laboratory of Bioorganic Phosphorus Chemistry and Chemical Biology, Department of Chemistry, Tsinghua University, Beijing, China

**Keywords:** PLK1, O-GlcNAc, Mitosis, Uterine carcinoma, Ubiquitination, IPed, immunoprecipitated, MS, mass spectrometry, O-GlcNAc, O-linked β-N-acetylglucosamine, OGT, O-linked β-N-acetylglucosamine transferase, PLK1, polo-like kinase 1, PTM, post-translational modifications, sceHCD, stepped collisional energy/higher-energy collisional dissociation, SUMO, small ubiquitin-related modifier, TCGA, The Cancer Genome Atlas

## Abstract

The O-linked β-N-acetylglucosamine (O-GlcNAc) transferase (OGT) mediates intracellular O-GlcNAcylation modification. O-GlcNAcylation occurs on Ser/Thr residues and is important for numerous physiological processes. OGT is essential for dividing mammalian cells and is involved in many human diseases; however, many of its fundamental substrates during cell division remain unknown. Here, we focus on the effect of OGT on polo-like kinase 1 (PLK1), a mitotic master kinase that governs DNA replication, mitotic entry, chromosome segregation, and mitotic exit. We show that PLK1 interacts with OGT and is O-GlcNAcylated. By utilizing stepped collisional energy/higher-energy collisional dissociation mass spectrometry, we found a peptide fragment of PLK1 that is modified by O-GlcNAc. Further mutation analysis of PLK1 shows that the T291A mutant decreases O-GlcNAcylation. Interestingly, T291N is a uterine carcinoma mutant in The Cancer Genome Atlas. Our biochemical assays demonstrate that T291A and T291N both increase PLK1 stability. Using stable H2B-GFP cells, we found that PLK1-T291A and PLK1-T291N mutants display chromosome segregation defects and result in misaligned and lagging chromosomes. In mouse xenograft models, we demonstrate that the O-GlcNAc–deficient PLK1-T291A and PLK1-T291N mutants enhance uterine carcinoma in animals. Hence, we propose that OGT partially exerts its mitotic function through O-GlcNAcylation of PLK1, which might be one mechanism by which elevated levels of O-GlcNAc promote tumorigenesis.

O-linked β-N-acetylglucosamine (O-GlcNAc) glycosylation occurs on Ser/Thr residues and mediates cellular signal transduction by crosstalk with phosphorylation and ubiquitination (1, 2). Currently, about 5000 proteins have been identified by proteomic or single-protein studies to be O-GlcNAcylated (3) and more continue to be discovered. O-GlcNAc transferase (OGT) has been found to regulate many biological processes, including transcription (4), immune response (5), neurodegeneration (6), metabolism (7), and cancer (8). In the case of tumor biology, OGT and O-GlcNAc are upregulated in many cancer types, including bladder cancer, breast cancer, and lung cancer (9).

Cell division is a fundamental biological process, in which one mother cell gives rise to two daughter cells. OGT depletion has long been found to impede cell cycle progression (10), while OGT overproduction leads to chromosome bridges (11). Conversely, the cell division machinery also regulates OGT. For instance, OGT protein abundance decreases during mitotic onset (12), and checkpoint kinase 1 (Chk1) phosphorylates OGT to stabilize OGT during cytokinesis (13). The reciprocal regulation between the cell cycle and OGT ensures the fidelity of mitosis (14, 15).

A proteomic study aiming to elucidate mitotic OGT substrates identified many O-GlcNAcylated proteins that function during spindle assembly and cytokinesis, the later stages of the cell cycle (11). One of the important OGT interactors is polo-like kinase 1 (PLK1). PLK1 is a mitotic master kinase regulating DNA replication, mitotic onset, spindle assembly, centrosome disjunction, chromosome segregation, spindle checkpoint, and cytokinesis (16, 17). As a pivotal mitotic kinase, PLK1 is subjected to a plethora of post-translational modifications (PTMs): its activation, symbolized by Thr210 phosphorylation, is mediated by the Aurora A kinase together with Bora (18); its inactivation, as indicated by Thr210 dephosphorylation, is modulated by the myosin phosphatase targeting subunit 1-protein phosphatase 1cβ complex (19). It is phosphorylated at Tyr217, Tyr425, and Tyr445 by the nonreceptor tyrosine kinase c-ABL to promote protein stability and activity in cervical cancer (20); it is deubiquitinated by ubiquitin-specific peptidase 16 to promote kinetochore localization for proper chromosome alignment in early mitosis (21); it is ubiquitinated at Lys492 by cullin 3-KLHL22 and thus removed from kinetochores to achieve stable kinetochore-spindle attachment (22). It is monomethylated at Lys209 and Lys413 by SETD6 to antagonize pThr210 and thus inactivated (23, 24); it is dimethylated at Lys191 by SET7/9 in early mitosis for accurate kinetochore-microtubule dynamics (25); it is also subject to small ubiquitin-related modifier (SUMO) modification at Lys492, which results in its nuclear import and increased protein abundance (26); and it is degraded by the anaphase-promoting complex/cyclosome during mitotic exit (27). Such multi-layered regulation showcases the intricate biological network needed to regulate correct localization, the rise and fall in protein abundance, and timely activation and inactivation of PLK1, resulting in successful mitosis. The role of PLK1 is not confined to mitosis: it is also implicated in cell motility, epithelial-to-mesenchymal transition, and cancer. Many targeted therapies are being developed to treat PLK1-associated diseases (28).

In an OGT-centered mitotic screen, PLK1 was found to colocalize with a subset of OGT (11). OGT overexpression reduces PLK1 transcripts and PLK1 protein abundance. But it does not alter PLK1-pThr210 levels or the localization pattern of PLK1 (11). Here, we present evidence that PLK1 is O-GlcNAcylated. Through stepped collisional energy/higher-energy collisional dissociation (sceHCD) mass spectrometry (MS), we found an O-GlcNAc–modified peptide. Mutagenesis studies demonstrate that the T291A and T291N mutations significantly downregulated O-GlcNAcylation levels. Mutations of T291A or T291N decrease PLK1 ubiquitination, resulting in increased PLK1 protein levels and misaligned or lagging chromosomes. Through The Cancer Genome Atlas (TCGA) and xenograft analysis, we further correlate our biochemical and cytological studies with pathological consequences: T291A and T291N mutations and PLK1 upregulation promote uterine carcinoma. Our work suggests that PLK1 O-GlcNAcylation is essential for dividing mammalian cells, and its aberration would lead to tumorigenesis.

## Results

### PLK1 is O-GlcNAcylated

**Figure 1:**
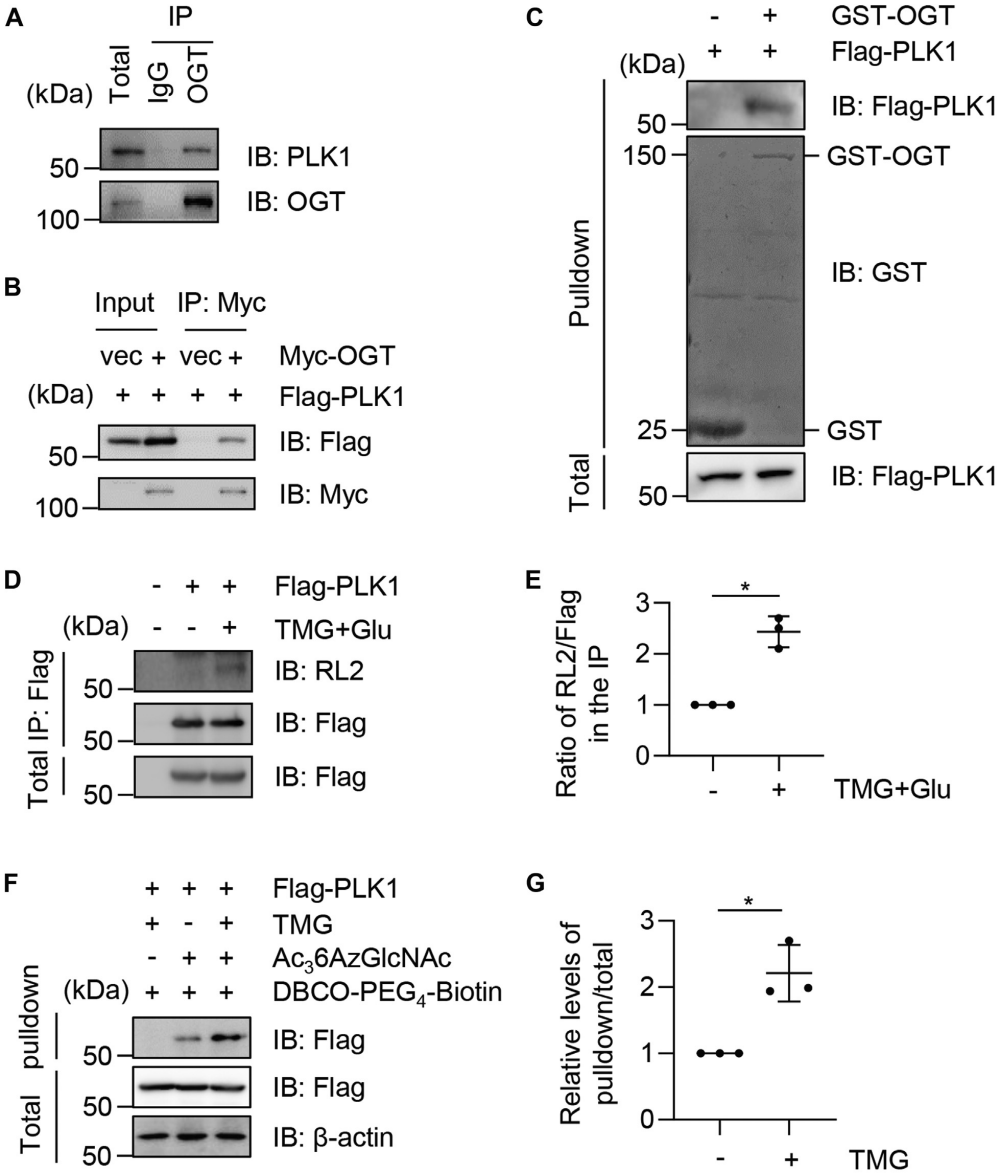
**PLK1 is O-GlcNAcylated.***A*, endogenous PLK1 interacts with OGT. *B*, cells were transfected with Flag-PLK1 and Myc-OGT plasmids, and the cell lysates were immunoprecipitated and immunoblotted with the antibodies indicated. *C*, cells were transfected with Flag-PLK1. Recombinant GST-OGT proteins were incubated with the cellular lysates, and GST pull-down experiments were carried out. *D*, cells were treated with the OGA inhibitor Thiamet-G (TMG) plus glucose as previously described (29). Cellular lysates were blotted with anti-O-GlcNAc antibodies RL2. ∗ indicates the O-GlcNAcylated band. *E*, quantitation of (D). ∗ indicates significant differences as determined by one-way ANOVA (*p* < 0.05). *F*, cells were transfected with Flag-PLK1, then treated with 200 μmol/l Ac_3_6AzGlcNAc or not treated and treated with 5 μmol/l TMG or not treated as previously described (30). *G*, quantitation of (*F*). ∗ indicates significant differences as determined by one-way ANOVA (*p* < 0.05). O-GlcNAc, O-linked β-N-acetylglucosamine; OGT, O-GlcNAc transferase; PLK1, polo-like kinase 1.

As Plk1 is an important mitotic kinase, and PLK1 has been reported to colocalize with OGT (11), we wondered whether OGT could directly O-GlcNAcylate PLK1. To this end, we first examined the potential biochemical interaction between OGT and PLK1. As shown in Fig. 1*A*, cell extracts were immunoprecipitated (IPed) with anti-OGT antibodies and the immunoprecipitates were immunoblotted (IBed) with anti-PLK1 antibodies. The two proteins coimmunoprecipitate. We also examined the interaction between overproduced proteins (Fig. 1*B*). Cells were transfected with Flag-Plk1 and Myc-OGT plasmids, then the cellular lysates were IPed with anti-Myc antibodies and immunoblotted with anti-Flag antibodies. Again, the overproduced proteins were found to associate. Recombinant GST-OGT proteins were also utilized. Cells were transfected with Flag-PLK1, and the cellular extracts were incubated with GST-OGT proteins (Fig. 1*C*). GST-OGT proteins could pull down Flag-PLK1 (Fig. 1*C*). We then examined PLK1 O-GlcNAcylation. As shown in Fig. 1*D*, WT Flag-PLK1 shows RL2 (an O-GlcNAc antibody) staining in the IB. Then, we enriched for O-GlcNAcylation with the treatment of the OGA inhibitor Thiamet-G together with glucose as described previously (29), and the RL2 band increased in intensity significantly. We also utilized click chemistry as previously described (30). Cells were transfected with Flag-Plk1 and incubated with Ac_3_6AzGlcNAc, then the lysates were incubated with DBCO-PEG_4_-Biotin. As shown in Fig. 1, *F* and *G*, the pull-down experiments further demonstrated that PLK1 is O-GlcNAcylated.

**Figure 2:**
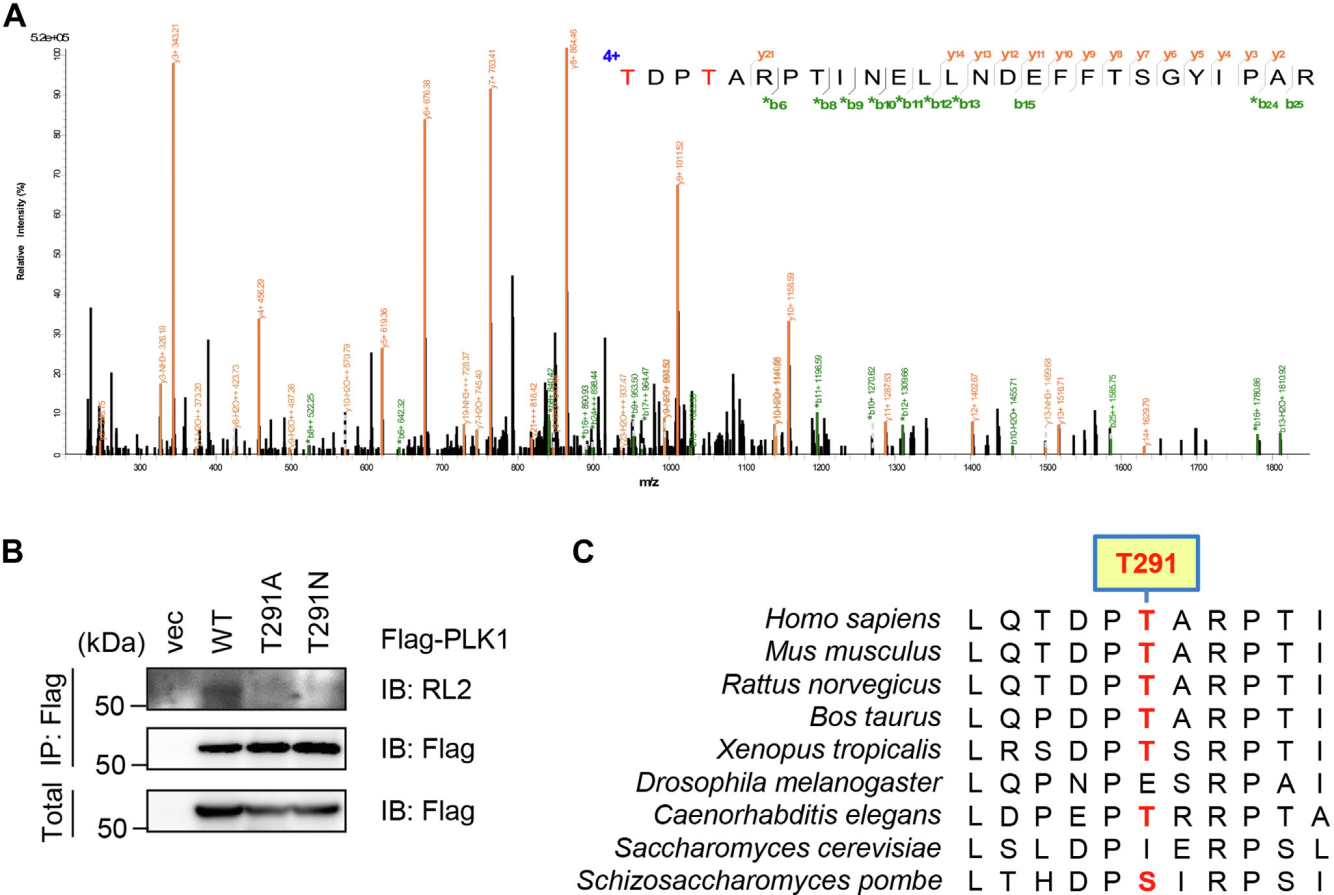
**The T291A and T291N mutations abolishes PLK1 O-GlcNAcylation.***A*, cells were transfected with Flag-PLK1 and enriched for O-GlcNAcylation. Anti-Flag immunoprecipitates were subject to stepped collisional energy/higher-energy collisional dissociation (sceHCD) mass spectrometry analysis. Results showed that Thr291 could be an O-GlcNAcylation site. ∗ indicates ions with neutral loss of OGT. *B*, cells were transfected with PLK1-WT, PLK1-T291A, or PLK1-T291N plasmids and the lysates were subjected to immunoprecipitation and immunoblotting assays. *C*, Thr291 of PLK1 is conserved in multiple species. OGT, O-GlcNAc transferase; PLK1, polo-like kinase 1.

Then, the Flag-PLK1–transfected cellular lysates were IPed, and the immunoprecipitates were subject to MS analysis (Fig. 2*A*). Electron-transfer dissociation or higher-energy collisional dissociation MS did not identify any sites (data not shown). Then, we conducted sceHCD MS, a method widely utilized for both N-glycopeptide analysis (31, 32) and O-glycosylation characterization (33). sceHCD identified a peptide modified by two O-GlcNAc residues and fragmentation suggested that Thr288 and Thr291 were modified (Fig. 2*A*). Mutation at Thr291 significantly reduced glycosylation of PLK1, suggesting that this site was modified and that mutation of this region impacted the ability of OGT to glycosylate other residues in PLK1. Therefore, we focused our study on Thr291. Incidentally, TCGA reveals that T291N is associated with uterine serous carcinoma/uterine papillary serous carcinoma (cbioportal.org). So, we also included T291N in our study. Both T291A and T291N mutants greatly diminished RL2 signals (Fig. 2*B*). Sequence alignment shows that PLK1 Thr291 is conserved in various organisms, but not in fly or budding yeast (Fig. 2*C*). Taken together, our results suggest that PLK1 is O-GlcNAcylated and the major modification site could be Thr291.

### PLK1 O-GlcNAcylation promotes ubiquitination

**Figure 3:**
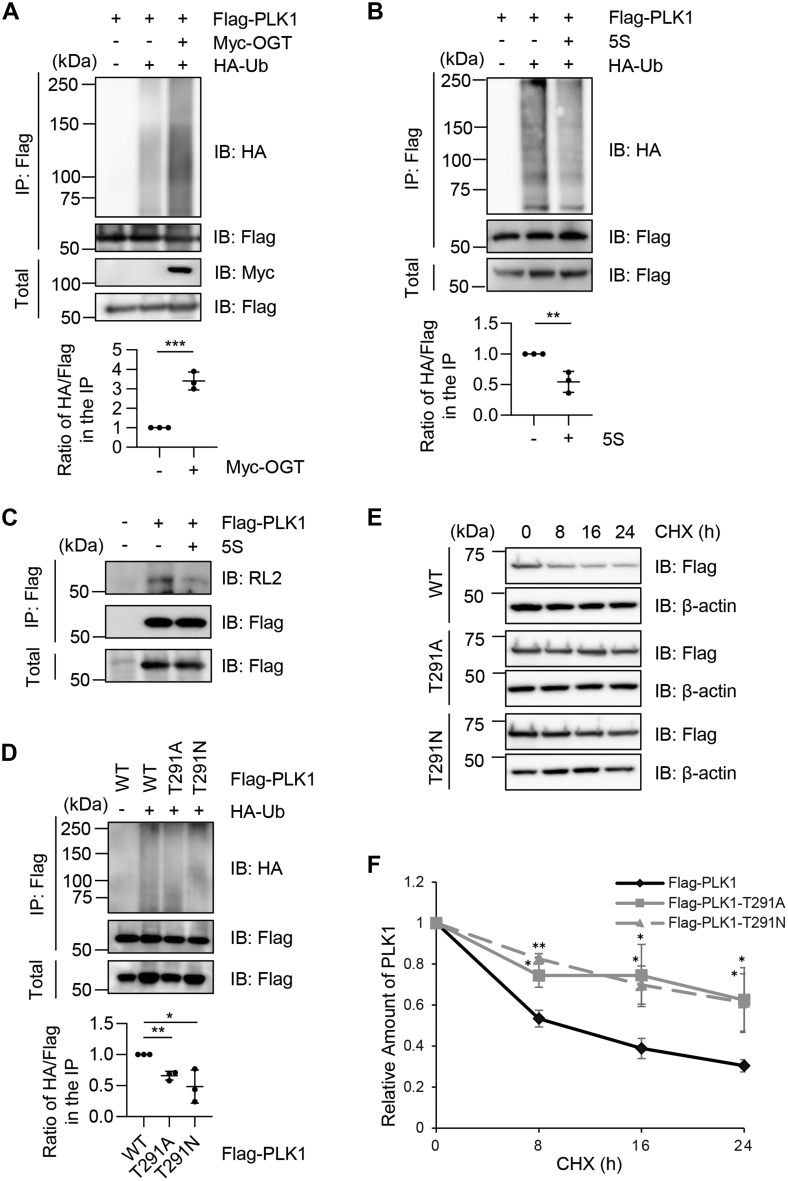
**PLK1 O-GlcNAcylation promotes ubiquitination.***A*, cells were transfected with Flag-PLK1, Myc-OGT, and HA-Ub. The lysates were immunoprecipitated with anti-FLAG antibodies and immunoblotted with the antibodies indicated. *B*, cells were transfected with Flag-PLK1 and HA-Ub, then treated with the OGT inhibitor Acetyl-5S-GlcNAc (5S) or not treated. *C*, cells were transfected with Flag-PLK1, then treated with 5S or not treated. *D*, cells were transfected with Flag-PLK1-WT, Flag-PLK1-T291A, Flag-PLK1-T291N, and HA-Ub. Quantitation was carried out with one-way ANOVA; ∗ indicates *p* < 0.05; ∗∗ indicates *p* < 0.01, ∗∗∗ indicates *p* < 0.001. *E* and *F*, cycloheximide (CHX) pulse-chase assays. Cells were transfected with PLK1-WT, PLK1-T291A, or PLK1-T291N, then treated with CHX for different durations. The quantitation is in (*F*). A two-way ANOVA test was used for statistical analysis. ∗ indicates *p* < 0.05; ∗∗ indicates *p* < 0.01. OGT, O-GlcNAc transferase; PLK1, polo-like kinase 1.

A previous study suggests that OGT overproduction reduces PLK1 protein abundance (11), so we examined PLK1 ubiquitination levels in the O-GlcNAcylation mutant. As shown in Fig. 3*A*, cells were transfected with Flag-PLK1, Myc-OGT, and HA-Ub plasmids. OGT overexpression markedly increased PLK1 ubiquitination levels. We also measured PLK1 ubiquitination using the chemical OGT inhibitor Acetyl-5S-GlcNAc (5S) (Fig. 3*B*), and PLK1 ubiquitination levels decreased upon OGT inhibition. We further verified that 5S decreased PLK1 O-GlcNAcylation (Fig. 3*C*). Then we used the O-GlcNAc–deficient mutants of T291A and T291N (Fig. 3*D*), and observed that the mutants downregulated ubiquitination levels compared with WT, suggesting that O-GlcNAcylation will increase PLK1 ubiquitination.

Then, we employed cycloheximide pulse-chase experiments (Fig. 3*E*). Cells were transfected with Flag-PLK1-WT, Flag-PLK1-T291A, or Flag-PLK1-T291N plasmids and treated with cycloheximide to inhibit new protein synthesis. The cellular lysates were collected at different time points to examine protein stability. As shown in Fig. 3, *E* and *F*, the O-GlcNAc-deficient T291A and T291N mutants increased protein half-life compared to WT, consistent with the ubiquitination assays. In sum, consistent with previous investigations (11), our biochemical results show that O-GlcNAcylation destabilizes PLK1, probably through enhanced ubiquitination.

### PLK1 O-GlcNAcylation promotes mitotic progression

**Figure 4:**
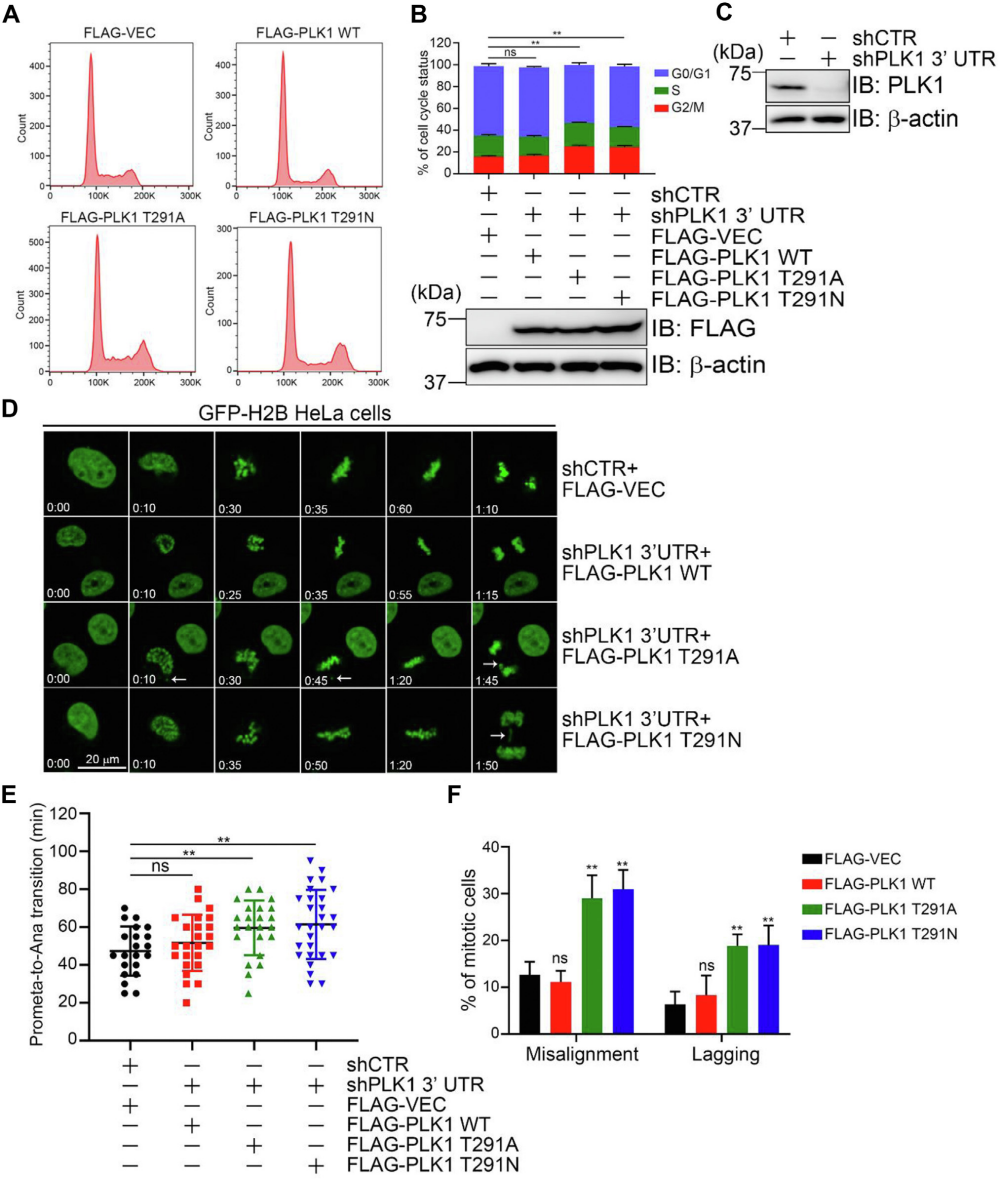
**Both T291A and T291N of PLK1 delay mitotic progression and induce chromosomal segregation defects.***A*–*C*, T291A and T291N of PLK1 delay mitotic progression. GFP-H2B HeLa cells stably expressing FLAG-vec, PLK1-WT, PLK1-T291A, or PLK1-T291N were constructed, where endogenous PLK1 was downregulated by shRNA targeting the 3′ UTR of *PLK1*. The expression of endogenous and exogenous PLK1 was analyzed by Western blots (*B* and *C*). Then, the cells were stained with propidium iodide (PI) and analyzed by flow cytometry (*A*) and quantified in (*B*) (upper panel). *D*–*F*, T291A and T291N of PLK1 induce chromosomal segregation defects. The cells in (*A*) were subjected to time-lapse imaging. Representative time-lapse images are shown with the acquisition time relative to the onset of mitosis (*D*). The arrows indicate misaligned chromosomes or lagging chromosomes. The durations of prometaphase to anaphase onset in each group were quantified in (*E*). The percentages of misaligned or lagging chromosomes were quantified in (*F*). Scale bar represents 20 μm. The data are presented as the mean ± SEM. Quantitation was carried out with a *t* test, ∗∗*p* < 0.01. ns, nonspecific. PLK1, polo-like kinase 1.

PLK1 is an instrumental mitotic kinase (34), so we assayed for mitotic defects of these O-GlcNAc mutants. We first constructed GFP-H2B HeLa cells stably expressing FLAG-vec, PLK1-WT, PLK1-T291A, or PLK1-T291N (Fig. 4*B*), in which endogenous PLK1 is depleted by shRNA targeting PLK1 (Fig. 4*C*). When the cell cycle profile was assessed with flow cytometry, the T291A and T291N mutants had a clear increase in G2/M cells compared with WT (Fig. 4, *A* and *B*).

Then, we analyzed chromosome segregation *via* time-lapse microscopy. The timing of cell cycle progression and phenotype of mitotic defects were monitored (Fig. 4*D*). Notably, the T291A and T291N mutants showed a prolonged prometaphase to anaphase transition (Fig. 4*E*) and misaligned or lagging chromosomes (Fig. 4*F*). These defects are reminiscent of the SUMO mutant of PLK1 (K492R) (26). We explored possible crosstalk between PLK1 O-GlcNAcylation and SUMOylation, but no correlation was found (data not shown). We also measured for pT210, and the T291A and T291N mutants also did not affect pT210 (data not shown). These results indicate that PLK1 O-GlcNAcylation, and possibly its resultant PLK1 degradation, is essential for a successful mitotic cell cycle.

### PLK1 O-GlcNAcylation inhibits uterine carcinoma

**Figure 5:**
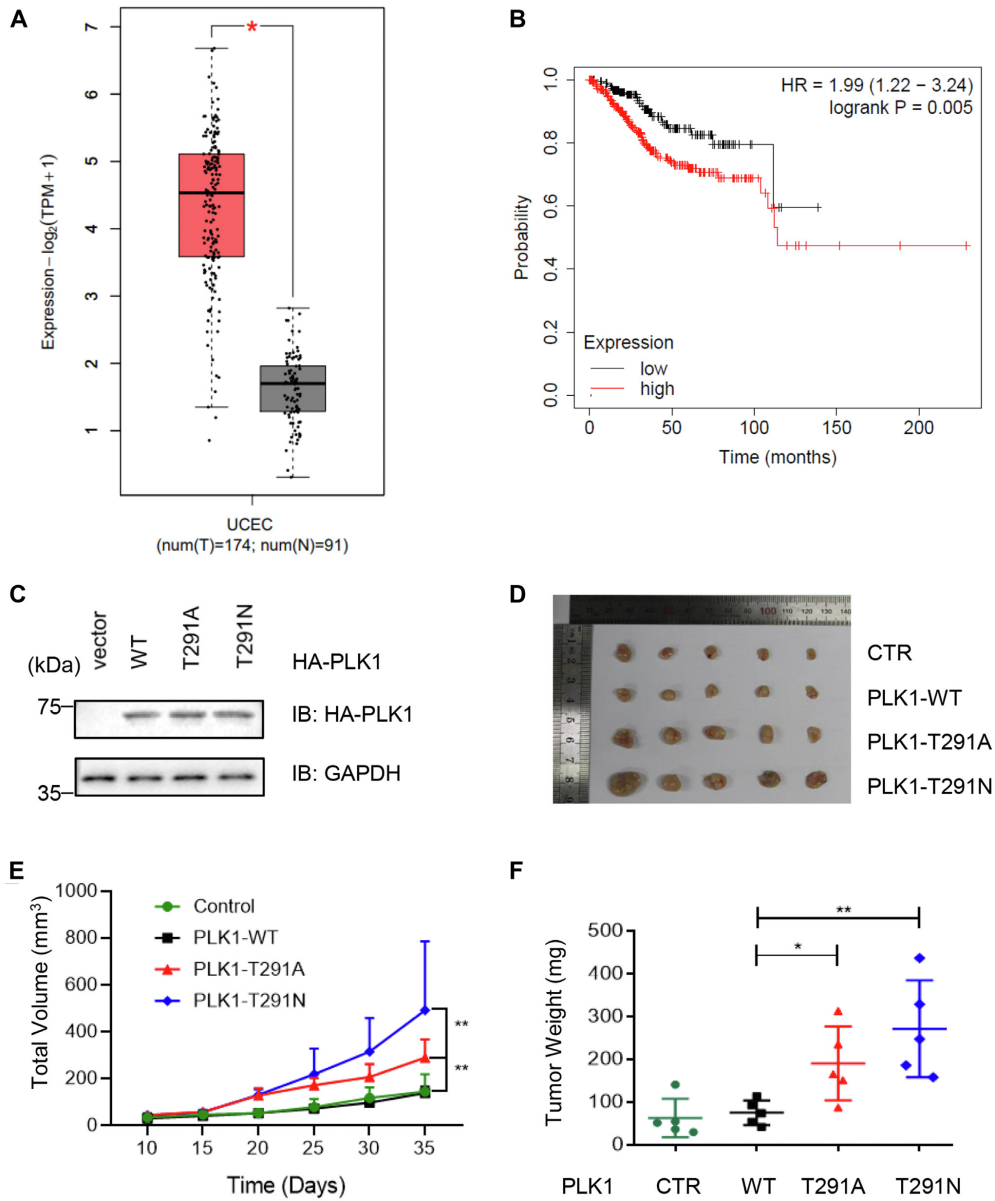
**PLK1 O-GlcNAcylation mutants induce uterine carcinoma in xenograft models.***A*, PLK1 mRNA levels in uterine corpus endometrial carcinoma (UCEC) samples from The Cancer Genome Atlas (TCGA). *B*, Kaplan–Meier survival curves of UCEC patients with high or low PLK1 expression levels (https://cistrome.shinyapps.io/timer/). *p*-value was calculated with the chi-square test. *C*, establishment of PLK1-WT and mutant stable cell lines in HeLa cells. *D* and *E*, xenografts in nude mice. PLK1-WT, PLK1-WT-T291A, and PLK1-WT-T291N cells were injected into nude mice. Tumors were photographed after 35 days. Tumor images are in (*D*), and tumor volumes are in (*F*). Quantitation was carried out with a *t* test, ∗*p* < 0.05; ∗∗*p* < 0.01. PLK1, polo-like kinase 1.

As T291N is a mutant involved in uterine carcinoma in TCGA, we investigated the correlation between PLK1 and uterine cancer. First, based on bioinformatics analysis, PLK1 mRNA levels in uterine corpus endometrial carcinoma samples from TCGA database were examined, and PLK1 overproduction was observed in cancer samples (Fig. 5*A*). Kaplan–Meier survival curves of uterine corpus endometrial carcinoma patients with high PLK1 expression levels were analyzed and showed a poorer prognosis (Fig. 5*B*) (https://cistrome.shinyapps.io/timer/). Second, we constructed stable PLK1-WT, PLK1-T291A and PLK1-T291N HeLa cells (Fig. 5*C*), and the PLK1 expression levels were comparable. Third, the cell lines were injected into nude mice and tumor growth was monitored (Fig. 5*D*). The T291A or T291N mice exhibited increased tumor volume and growth compared to WT (Fig. 5, *E* and *F*), suggesting that chromosome mis-segregation and other mitotic defects might underlie uterine cancer growth.

## Discussion

In this study, we found that the mitotic master kinase PLK1 is O-GlcNAcylated and further identified a major O-GlcNAcylation site, Thr291. Thr291 O-GlcNAcylation is instrumental for mitotic progression and its aberration contributes to uterine carcinoma in xenograft models and possibly in humans. Our findings are in line with previous observations that OGT partially colocalizes with PLK1 and negatively regulates PLK1 protein stability (11).

Many proteomic profiling studies have shown negative crosstalk between O-GlcNAcylation and ubiquitination (35), that is, O-GlcNAcylated proteins tend to be more stable. In the case of PLK1, however, our data show that O-GlcNAcylation promotes ubiquitination and subsequent degradation. We sought to identify the underlying mechanisms by examining the possible effect on the interaction between PLK1 and Cdh1 (also known as Fzr1), as PLK1 is degraded during mitotic exit by anaphase-promoting complex/cyclosome (27). But no alteration was observed in the O-GlcNAc–deficient T291A or T291N mutants. It is possible that O-GlcNAc might interfere with the association of PLK1 and other E3 ligases.

We also tried to examine the possible crosstalk between O-GlcNAc with other PTMs. Structurally, PLK1 comprises an N-terminal kinase domain (amino acid 53-303), a D-box (337-345), and a C-terminal Polo-box domain (345-603) (36). Thr291 thus resides in the kinase domain. But neither T291A nor T291N affects the activation phosphorylation of pThr210 (data not shown), consistent with previous results (11). SUMOylation at Lys492, another PTM that modulates PLK1 stability (26), was also examined. PLK1 O-GlcNAcylation does not show crosstalk with SUMOylation either (data not shown), probably due to the long distance between the two PTM sites.

Investigators have previously discovered that OGT depletion hampers cell cycle progression, and many OGT substrates during cell division have been identified (11, 37, 38), suggesting that OGT has various roles in mitosis. Our work here demonstrates that PLK1, the master mitotic kinase, is O-GlcNAcylated, and if this does not occur, it leads to mitotic defects. PLK1 is upregulated in many cancer types, and PLK1 inhibitors have been actively pursued as an anticancer therapy (39). As a “druggable target”, PLK1 inhibitors such as BI2536 and BI6727 (volasertib) were developed targeting its kinase domain, with volasertib reaching phase III trials (39). Our new finding of Thr291 O-GlcNAcylation reveals a new layer of regulation of PLK1 and may provide extra targets for designing next-generation inhibitors.

## Experimental procedures

### Cell culture, antibodies, and plasmids

HeLa cells were purchased from ATCC. *OGT* plasmids and OGT antibodies were described before (13). PLK1 antibodies were Santa Cruz, sc-17783; RL2 antibodies were Abcam, AB2739. *PLK1* mutant plasmids were generated using specific primers (sequences available upon request) following the manufacturer's instructions (QuickChange II, Stratagene). sh*PLK1* UTR: CCGGAGCTGCATCATCCTTGCAGGTCTCGAGACCTGCAAGGATGATGCAGCTTTTTT.

### Immunoprecipitation and immunoblotting assays

Immunoprecipitation and immunoblotting experiments were performed as described before (40). The following primary antibodies were used for immunoblot: anti-HA (1:1000), anti-FLAG M2 (Sigma) (1:1000), anti-Myc (1:1000), anti-PLK1 (1:1000). Peroxidase-conjugated secondary antibodies were from Jackson Immuno Research. The ECL detection system (Amersham) was used for immunoblot. LAS-4000 was employed to detect signals and quantitated by the Multi Gauge software (Fujifilm). All western blots were repeated for at least 3 times. Chemical utilization: acetyl-5S-GlcNAc (5S-G) (OGT inhibitor) was used at 100 μM (prepared at 50 mM in DMSO) for 24 h.

### Bioorthogonal chemistry assays

Bioorthogonal chemistry experiments were performed as described before (30). Cells were treated with 200 μmol/l Ac_3_6AzGlcNAc for 24 h. Collected cells were lysed with 150 mM lysis buffer (150 mM NaCl, 1 M Tris (pH7.5), 0.5 M EDTA, and 10% NP-40) containing a protease inhibitor cocktail (Roche) for 1 h at 4 °C. Next, cell lysates were cleared using centrifugation (4 °C; 12,000 rpm; 10 min). The supernatant reacted with 50 μmol/l DBCO-PEG_4_-Biotin from Duyouyou Biotechnology, 8 mmol/l urea, 10 mmol/l Hepes (pH 7.9), and Halt protease & phosphatase inhibitor cocktail (100 × ) from Thermo Fisher Scientific, then the pull-down complex isolated by streptavidin-coupled beads was subjected to Western blotting analysis.

### sceHCD mass spectrometry

For identification of O-GlcNAcylation by MS, PLK1 proteins isolated by gel electrophoresis were digested with trypsin (Promega) in 100 mM NH_4_HCO_3_ pH 8.0. The LC-MS/MS analysis was performed on an Easy-nLC 1000 II HPLC (Thermo Fisher Scientific) coupled to a Q-Exactive HF mass spectrometer (Thermo Fisher Scientific). Peptides were loaded on a precolumn (75 μm ID, 6 cm long, packed with ODS-AQ 10 μm, 120 Å beads from YMC Co, Ltd) and further separated on an analytical column (75 μm ID, 12 cm long, packed with Luna C18 1.9 μm 100 Å resin from Welch Materials) using a linear gradient from 100% buffer A (0.1% formic acid in H_2_O) to 30% buffer B (0.1% formic acid in acetonitrile), 70% buffer A in 75 min at a flow rate of 200 nl/min. The top 20 most intense precursor ions from each full scan (resolution 120,000) were isolated for higher-energy collision dissociation MS2 {resolution 15,000; three-step normalized collision energy (25, 27, 30)} with a dynamic exclusion time of 60 s. Precursors with a charge state of 1+, 7+ or above, or unassigned, were excluded.

The software pFind 3 (41, 42; http://pfind.org/software/pFind/index.html) was used to identify O-GlcNAcylated peptides by setting a variable modification of 203.0793 Da at S, T. The mass accuracy of precursor ions and that of fragment ions were both set at 20 ppm. The results were filtered by applying a 1% false discovery rate cutoff at the peptide level and a minimum of 1 spectrum per peptide. The MS2 spectra were annotated using pLabel (43).

### Cell cycle profile analysis

GFP-H2B stably expressing HeLa cells were harvested with trypsin, fixed in 75% ice-cold ethanol overnight, and stained with propidium iodide at room temperature for 30 min, followed by DNA content analysis using a CytoFLEX flow cytometer (Beckman Coulter). The cell cycle distribution was analyzed using FlowJo software (https://www.flowjo.com/).

### Time-lapse microscopy assays

HeLa cells stably expressing GFP-H2B were infected with lentiviral plasmids carrying FLAG-VEC, PLK1 WT, T291A or T291N constructs. Endogenous PLK1 was knocked down by shRNA targeting the 3′ UTR on PLK1. For all time-lapse recordings, the culture dish was placed in a microincubator to maintain proper environmental conditions (37 °C). All images were acquired using an Andor Dragonfly confocal microscope.

### Mouse xenograft analysis

For xenograft assays, 5 X 10^5^ WT or mutant cells were resuspended in Matrigel (Corning) and then injected into the flanks of nude mice (6–8 weeks old). The tumor volumes were measured every 5 days. At 35 days after the injection, tumors were dissected. The mice were obtained from the Beijing SPF Biotechnology Co, Ltd [Certification NO. SCXK (Jing) 2019-0010]. All animal work procedures were approved by the Animal Care Committee of the Capital Normal University (Beijing, China).

## Data availability

The mass spectrometry proteomics data have been deposited to the ProteomeXchange Consortium (http://proteomecentral.proteomexchange.org) *via* the iProX partner repository (44) with the dataset identifier PXD036141.

## Conflict of interest

The authors declare that they have no conflicts of interest with the contents of this article.
